# Comparing frequencies of adolescent suicide attempters pre- and during COVID-19 pandemic school terms

**DOI:** 10.1186/s12888-024-05823-y

**Published:** 2024-05-17

**Authors:** Rebeca Gracia-Liso, Maria J. Portella, Elena Pujals-Altés, Joaquim Puntí-Vidal, Marta Llorens, Montserrat Pàmias, Marc Fradera Jiménez, Itziar Montalvo Aguirrezabala, Diego J. Palao

**Affiliations:** 1 Department of Mental Health, Parc Taulí-University Hospital of Sabadell, Barcelona, 08208 Spain; 2 Department of Psychiatry and Forensic Medicine, Autonomous University of Barcelona (UAB), Barcelona, 08193 Spain; 3 Institut d’Investigació i Innovació Parc Taulí I3PT-CERCA, Unitat de Neurociència Traslacional I3PT-INc, Sabadell, 08208 Spain; 4 Sant Pau Mental Health Research Group, Institut de Recerca Sant Pau (IR Sant Pau), Barcelona, 08041 Spain; 5 Department of Psychiatry, Hospital de la Santa Creu i Sant Pau, Barcelona, 08041 Spain; 6 Centro de Investigación Biomédica en Red de Salud Mental (CIBERSAM), Madrid, 28029 Spain; 7 Child and Adolescent Psychiatry and Psychology Department, Hospital Sant Joan de Déu of Barcelona, Barcelona, 08950 Spain

**Keywords:** Adolescent suicide, COVID-19, Suicide attempt, Seasonality of suicide, Triggers of suicide

## Abstract

**Background:**

The COVID-19 pandemic had and still have a major impact on adolescent mental health and consequently on suicidal behavior. However, few studies have investigated whether the pandemic has changed the patterns and the triggers of suicidality peaks in adolescents, e.g., seasonal patterns or family conflicts. We hypothesized that the pandemic modified suicidality rates: an increment of suicide attempts would be observed in the first semester of the academic year during COVID-19 pandemic compared to the same period of previous academic year; and the precipitating factors would be more related to social stressors during the pandemic school year.

**Methods:**

A retrospective cross-sectional study was conducted to assess the precipitating factors, also including school-related factors and cognitive skills, of adolescent suicide attempters occurred in the first and second semesters of the year before the pandemic lockdown (study periods 1 and 2) and the year after (study periods 3 and 4).

**Results:**

The sample consisted of 85 adolescents aged between 12 and 17 recruited consecutively from March 2019 to March 2021 at emergency ward because of suicide attempt. Forty-eight adolescents (55.3% of the sample) were attended before the lockdown (pre-pandemic group) and 38 (44.7%) the year after. The results showed a higher proportion of female suicide attempters in period 4 (Sept 2020-Feb 2021) respect to period 3 (Mar 2020-Aug 2020), i.e., pandemic semesters compared with the increment observed between period 2 and 1 (prepandemic semesters; Fisher’s exact test = 4.73; *p* = 0.026). The multinomial regression models showed a significant effect in the frequency of adolescents who attempted suicide (ratio χ^2^ = 15.19, *p* = 0.019), accounted by the differences between period 4 (Sept 2020-Feb 2021) and period 1 (Mar 2019-Aug 2019), with depressive symptoms being a significant contributing factor (Exp(b) = 0.93; *p* = 0.04). Additionally, social triggers and age were found to be significant risk factors for suicide attempts in the first semester of the pandemic academic year (period 4) compared to the same semester of the pre-pandemic year (period 2; Exp(b) = 0.16, *p* = 0.01, and Exp(b) = 1.88, *p* = 0.006, respectively).

**Conclusions:**

During the pandemic, the decrement between first and second semester of the number of females attempting suicide was more pronounced than in the prepandemic school year -though this findings lacked statistical power due to very limited sample size-. Change in the frequency of adolescent attempting suicide in the different school periods was associated with greater severity of depressive symptoms. Social relations in back-to-school after the lockdown were also associated with the number of adolescents attempting suicide.

## Background

Many studies have pointed out the consequences of social isolation measures decreed by governments to prevent the spread of the COVID-19 on mental health of the population [1, 2] specially in adolescents [3], incrementing the prevalence of mental disorders, particularly anxiety and depression [4–6], but also of suicidal behaviors [7]. The closure of schools and the limitation of social activities has been interpreted as one of the causes of such increments [8]. Some studies further suggest that the COVID-19 pandemic and the isolation measures have contributed to change incidence rates of suicide behaviors [9] due to what some authors have called “the perfect storm“ [10] i.e., with an increase of risk factors for suicide and a decrease of protective factors (e.g., reduced access to health services) [11]. A recent study carried out in Catalonia showed that during the first months of the pandemic there was a decrease in suicidal behaviors that was reverted by a much higher increase the following months. Moreover, it was observed that this increase occurred at the expense of females under 18 years of age [12]. Specifically, a particularly prominent increase in suicidal behavior was observed in adolescent girls after 6 months from the onset of the pandemic, coinciding with the reopening of schools [13]. The COVID-19 pandemic has also brought about that adolescents who attempted suicide during the pandemic were diagnosed with new-onset psychiatric conditions, predominantly depression and anxiety [14] [22].

In this regard, the seasonality of suicide is a phenomenon that has been extensively studied. Many studies have shown an association between seasonality and suicide rates, mainly in adults, although it is unclear the reason of such association [15]. Some of the theories propose changes in sunlight, changes in sleep patterns, and serotonin production [16]. In general, most studies have found that suicide rates are higher in spring and summer compared to autumn and winter [17–20]. By contrast, research conducted with adolescents show that suicidal behavior is marked by school periods, with higher prevalence of suicidal attempts in spring and autumn and lower in summer [21, 22].

It is known that suicide is commonly attempted after an acute trigger in vulnerable individuals [23], family conflicts and academic stressors -including exam stress and peer rejection- [24]. Therefore, the aim of the present study is to find out whether return to school after months of confinement was a significant risk factor for attempting suicide among adolescents. We hypothesized that there would be a significant rise in the number or adolescents attempting suicide during the first semester of 2020–2021, consistent with previous research. Furthermore, we anticipated that the frequency of young suicide attempters during the school semesters prior to and during the pandemic would be influenced by several factors including depressive symptoms, precipitating factors, sex, and age.

## Methods

### Procedure

This is a secondary analysis of a previous work comparing the characteristics of adolescents who attempted suicide during the COVID pandemic with those who attempted suicide the year before the lockdown. A retrospective cross-sectional study was designed with adolescents who were referred to the Child and Adolescent Mental Health Service of a General Hospital of Catalonia (northwest region of Spain) from the emergency department after attempting suicide [14, 23].

The study was initially designed to assess risk factors for suicide in adolescents, but the spread of COVID-19, offered the chance to explore risk factors before and during the pandemic in this age group. The recruitment of the sample was carried out from March 2019 to March 2021. Adolescents who were referred to the child and adolescent mental health service after attempting suicide were invited to participate in the study, and the assessment was performed by a clinician (psychiatrist or psychologist). Inclusion criteria were: 1) adolescents aged 12–17 (inclusive) referred to the Child and Adolescent Mental Health Department after performed a suicide attempt. Exclusion criteria: (1) having a cognitive or neuropsychological impairment preventing assessment; (2) refusing to participate in the study; (3) language barriers. To demonstrate the hypotheses of the study, four study periods were defined: period 1: from March 2019 to August 2019 (2nd school-semester pre-pandemic); period 2: from September 2019 to February 2020 (1st school-semester pre-pandemic); period 3, from March 2020 to August 2020 (2nd school-semester pandemic; period 4, from September 2020 to February 2021 (1st school-semester pandemic). First semester corresponds to the beginning of the academic year and the second semester corresponds to the end of the school year and summer holidays.

The following variables were collected: age, sex (in two categories, males and females based on assigned sex at birth) and precipitating factors of suicide attempt as referred by the participants by choosing among the following options: related to social relationships, related to intimate relationships, related to family relationships, related to academic performance, or related to emotional management. The assessment of academic performance from the current academic year was requested to the participants and their legal guardians, and it was based on the results of their school grades, classified as excellent (mean grade higher than 8.5 out of 10), average (mean grade between 5 and 8.5 out of 10), or low (mean grade lower than 5 out of 10).

### Instruments

To assess the cognitive performance of the participants, the Wechsler Intelligence Scale for Children Fifth Edition (WISC-V) [25] was used with children aged 12–16, and the Wechsler Adult Intelligence Scale Third Edition (WAIS-III) [26], was used with adolescents aged 17. To assess depressive symptoms the Children’s Depression Inventory (CDI) by Kovacs [27] was used for children under 16 years old, and the Beck’s Depression Inventory (BDI) [28] for participants aged above 16. Both measures are highly correlated and have shown to be good instruments to screen depressive symptoms in adolescents [29]. Therefore, scores on the two questionnaires were merged to obtain a single variable (depressive symptoms) using the median approach -compute command in IBM SPSS that delivered a median-based value per each subject.

### Ethical considerations

The study complied with the internal regulations of the hospital’s ethics committee. All procedures were performed in accordance with the ethical principles set out in the Declaration of Helsinki 1995 (last version, Fortaleza 2013) [30]. All participants (parents and adolescents) gave their written informed consent after receiving the information regarding the study and its objectives, as well as assurances of confidentiality and personal data protection. Participation in the study was not remunerated.

### Data analysis

Normal distribution was checked for quantitative variables by means of skewness and kurtosis. Parametric and non-parametric tests were used for quantitative -and normally distributed variables- and qualitative or non-normally distributed variables, respectively. Descriptive analyses of qualitative variables were analyzed with chi-square or Fisher’s exact test; the number of participants and their percentage (n, %) were used for each category. Comparative analyses of the quantitative variables were carried out between the four study periods by means of two-way ANOVA or MANOVA (sex by study period). Multinomial regression models were used to estimate predictors of the frequency of adolescent suicide attempters in the 4 study periods. All statistical analyses were performed using the IBM SPSS software (BM Corp. Released 2017. IBM SPSS Statistics for Windows, Version 25.0. Armonk, NY: IBM Corp.). Statistical significance threshold was set at *p* < 0.05.

## Results

Table 1 displays the characteristics of the 85 participants divided into the four study periods. There were no significant differences in sex between the four groups (Fisher’s exact test = 0.004; *p* = 0.9), while the two-way ANOVA revealed no significant sex by study period interaction (F = 0.88; *p* = 0.7), but significant differences in age (study- period main-effect, F = 4.22; *p* = 0.008) where adolescents of period 4 were the youngest. Almost a 60% of adolescents had a low school performance and there were no significant differences among the four periods (Fisher’s exact test = 0.43; *p* = 0.5). Neither there were significant differences in intellectual ability nor in specific cognitive functioning (MANOVA omnibus; F = 1.08; *p* = 0.4). Depressive symptoms were similar in the all the study periods (F = 0.69; *p* = 0.6). The self-reported precipitating factors of the suicide attempt showed no statistically significant differences among the four periods (χ^2^ = 13.12; *p* = 0.4).

**Table 1:** Demographics, cognitive, clinical, school-related variables and precipitating factors of the participating adolescents with a suicide attempt, divided via the four study periods. *P*-values of qualitative variables correspond to chi-square statistic of Fisher’s exact test while those of quantitative variables, to univariate effects of the 2-way ANOVA or MANOVA (sex by study periods)

		Prepandemic Periods		Pandemic Periods		
Variables	Total (*n* = 85) (100%)	Period 1 (March19-Aug19) (*n* = 16) (18.8%)	Period 2 (Sep20- Feb20) (*n* = 31) (36.5%)	Period 3 (March20-Aug20) (*n* = 6) (7.1%)	Period 4 (Sep20-Feb21) (*n* = 32) (37.6%)	*p*-value
Age (mean ± SD)	15.14 **±** 1.42	15.00 **±** 1.15	15.74 **±** 1.18	15.50 **±** 1.56	14.56 **±** 1.42	**0.008**
Sex (% fem)	72(84.7%)	14(87.5%)	26(83.9%)	4(66.7%)	28(87.5%)	0.612
School performance						
Low	50(58.8%)	11(68.8%)	18(58.1%)	4(66.7%)	17(53.1%)	0.452
Average	27(31.8%)	5(31.3%)	8(25.8%)	1(16.7%)	13(40.6%)	
Excellent	8(9.4%)	0(0.0%)	5(16.1%)	1(16.7%)	2(6.3%)	
Cognitive performance measured with WISC-V						
WISCV-IA	95.99 **±** 16.20	89.19 **±** 13.42	95.10 **±** 19.14	102.0 **±** 14.96	99.94 **±** 13.26	0.094
WISCV-VCI	98.22 **±** 15.91	90.19 **±** 16.54	97.00 **±** 18.53	103.00 **±** 8.63	102.91 **±** 11.85	0.060
WISCV-VSI	101.35 **±** 16.97	98.88 **±** 11.93	96.26 **±** 19.97	111.60 **±** 10.47	106.63 **±** 16.87	0.059
WISCV-RFI	96.76 **±** 14.98	92.56 **±** 11.46	95.06 **±** 18.52	104.60 **±** 15.61	99.35 **±** 11.93	0.269
WISCV-WMI	93.06 **±** 15.42	86.19 **±** 11.93	91.45 **±** 16.76	98.40 **±** 18.71	97.35 **±** 14.13	0.087
WISCV–PSI	98.51 **±** 12.48	95.13 **±** 13.59	99.10 **±** 13.79	95.60 **±** 16.68	100.13 **±** 9.80	0.573
Precipitating factors related to Suicide Attempt						
Social relationships	17(20.0%)	3(18.8%)	2(6.5%)	1(16.7%)	11(34.4%)	0.177
Intimate relationships	11(12.9%)	2(12.5%)	4(12.9%)	1(16.7%)	4(12.5%)	
Family relationships	38(44.7%)	9(56.3%)	13(41.9%)	3(50.0%)	13(40.6%)	
Academic performance	8(9.4%)	0(0.0%)	4(12.9%)	1(16.7%)	3(9.4%)	
Emotional management	11 (12.9%)	2(12.5%)	8(25.8%)	0(0%)	1(3.1%)	
Screening depression measured by CDI and BDI						
Merged screening depression	26.31 ± 10.57	22.00 ± 8.57	27.67 ± 12.81	23.50 ± 6.56	27.77 ± 9.35	0.246

WISC V = Wechsler Intelligence Scale for Children; IA = intellectual ability; VCI = Verbal Comprehension Index; VSI = Visual Spatial Index; RFI = Fluid Reasoning Index; WMI = Working Memory Index; PSI = Processing Speed Index. Results in bold are statistically significant (*p* < 0.05). Values represent mean ± SD, otherwise specified

As can be seen in Table 2, the analysis performed by sex revealed higher number of suicide attempters were attended to in the emergency room in periods 2 and 4 (1st semesters) and with a higher frequency in girls. The difference between first and second semesters was more pronounced in the pandemic year (Fisher’s exact test = 4.73; *p* = 0.026).

**Table 2:** Number of suicide attempters by semester of the academic school year. *P*-values of qualitative variables correspond to chi-square statistic of Fisher’s exact test

Variable	First Semester of School Academic Year	Second Semester of School Academic Year	*p*-value
Prepandemic global (girls + boys, *n* = 47)	31(66.0%)	16(34.0%)	0.056
Pandemic global (girls + boys, *n* = 38)	32(84.2%)	6(15.8%)	
Prepandemic girls (*n* = 40)	26(65.0%)	14(35.0%)	**0.026**
Pandemic girls (*n* = 32)	28 (87.5%)	4(12.5%)	
Prepandemic boys (*n* = 7)	5(71.4%)	2(28.6%)	0.66
Pandemic boys (*n* = 6)	4(66.7%)	2(33.3%)	

Results in bold are statistically significant (*p* < 0.05)

Two independent multinomial regression models to predict frequencies of suicide attempts per period had to be run because unexpected singularities in the Hessian matrix were encountered when all variables related with the hypothesis were included. Therefore, one model included as predictors depressive symptoms, sex and age, while the other model included precipitating factors, sex and age. The reference category for the models was Period 4 as it was hypothesized to have the highest frequency. Regarding the models fitting information, the ratio chi-squared test for the first model was 15.19 (*p* = 0.019) and for the second one, 29.73 (*p* = 0.04), respectively. Estimates for the first model showed a significant effect of depressive symptoms in Period 1 respect Period 4 (Exp(b) = 0.932, *p* = 0.04) with higher frequency associated with greater severity, and a significant effect of age in Period 2 respect to Period 4 (Exp(b) = 1.85, *p* = 0.005) with higher frequency associated with younger adolescents. Sex was not significant in any estimation. Estimates for the second model revealed a significant effect of precipitating factors and age in Period 2 respect to Period 4 (Exp(b) = 0.16, *p* = 0.01); (Exp(b) = 1.88, *p* = 0.006), respectively, with higher frequency associated with social stressors and younger age.

## Discussion

Our findings showed that the decrement in the number of adolescent suicide attempters between first and second semesters in the pandemic year was more pronounced compared to the drop observed between school terms in the prepandemic year. These different frequencies during the four study periods were significantly associated with presence of depressive symptoms, social precipitating factors, and age. Unexpectedly, sex was not a significant factor in the multinomial models, which could suggest more complex interactions to explain the increment of suicide attempts.

The different frequencies of female suicide attempters were not accounted by an increase of suicide attempts in the first school term of the pandemic year but by a significant decrease during the second semester of the pandemic. These findings partly align with Ridout and colleagues’ research, which reported a significant increase in suicide-related emergency department visits among girls between September and December 2020, following a decrease in the early months of the COVID-19 pandemic [31]. Other authors, as for instance Yard et al., reported a significant rise in Emergency Department visits for self-harm attempts among girls aged 13–17 years after the lifting of confinement, particularly during winter 2021. This difference was not observed in boys of the same age or in the adult population [32] Some studies have reported seasonal trends in suicidality among adolescents, with an increase in suicide attempts and ideation at the beginning of the school year or after a long break. Carbone et al. analyzed data from the National Emergency Department Sample of the United States and found a clear seasonality trend for children and adolescents [33], while a similar study in Japan reported higher suicide frequency among middle and high school students at the start of the academic year or the fall semester [34]. Some authors suggest that the COVID-19 pandemic has exacerbated this trend in adolescent girls. However, our results reveal that there was a decrease during the period of strict confinement and summer vacations, which also coincide with less school-related stressors and social demands. Subsequently, with the reopening of schools, an increase of female suicide attempters attending the Emergency room was observed although less pronounced than these previous works. Differences with boys in this regard, have been described by Magson et al. reporting that girls showed more internalizing symptoms both at the beginning of school closure and after reopening, while boys did not display these changes [35]. Girls are more likely to have internalizing problems, particularly depression, during adolescence [36], and this difference has become more pronounced since the pandemic, suggesting that girls may be more sensitive to the social isolation caused by pandemic prevention measures.

The first model showed that the frequency of suicide attempts during the first school semester of the pandemic was significantly associated with depressive symptoms, with a higher frequency being observed in those with more symptoms. This finding is consistent with a previous study that found that adolescents who attempted suicide during the pandemic had more diagnoses of depression than those who did so before the pandemic [13]. Other studies have also shown an increase in depressive disorders among adolescents during the COVID-19 pandemic [5, 6, 37–41]. For instance, Evensen et al. reported an increase in mental health consultations for anxiety and depression during the fall of 2020 and in 2021, in addition to the already observed increases in the years prior to the pandemic [42].

The second model revealed that the frequency of suicide attempts during the first school semester of the pandemic was significantly associated with precipitating factors and age, with higher frequency being observed in those who experienced social stressors and in younger adolescents. Research has pointed out the consequences of school closure during the pandemic on the social development of adolescents, such as feelings of loneliness [43]. Loneliness during adolescence is a risk factor for mental health problems, including self-harm and suicidal ideation [44]. School routines are important coping mechanisms for adolescents with mental health problems, and school is an essential social environment for developing social cognition and understanding other people’s emotions [45]. Social deprivation during adolescence, including loneliness, is a risk factor for developing affective disorders [46]. The prolonged closure of schools and social isolation during the pandemic may have affected the development of adolescents’ social behavior, and the return to social life after months of isolation may have been experienced as an acute stressor for suicide attempts.

In our study, age was found to influence the frequency of suicide attempts during the first semester of the pandemic compared to pre-pandemic, with younger adolescents being more likely to attempt suicide. This finding is consistent with previous research that has warned about the increase in suicide attempts in adolescents, particularly younger girls [47]. Some protective factors for adolescent mental health during the pandemic have been identified, such as healthy parent-child relationships, communication, maintaining routines, and feeling connected to friends [4, 48]. Feeling a sense of social belonging can reduce the risk of psychopathology in adolescents [49]. Social group affiliation has also been shown to be a preventive factor for suicidal behavior in children and adolescents during the pandemic [50].

### Study limitations

The main limitations of this study include, first, the small sample size, which limits the statistical potential of the results, especially in the period 3. Second, the sample was obtained from a clinical population attending an emergency department after a suicide attempt which limit the generalization of the findings. Third, instruments assessing depressive symptoms were self-administered, meaning that results could be biased by a tendency among adolescents to maximize or minimize them. Fourth, due to statistical artifacts, two different multinomial models had to be run, and the independent effect of depressive symptoms and type of trigger could not be analyzed together as initially hypothesized. Fifth, no details of severity and intention of suicide attempts were included in the analyses or no validated instrument was used to assess the precipitating factors. Sixth, the study lacks a genuine gender perspective, which is very relevant because rates of suicide tend to be higher in non-binary individuals, as the analyses were stratified by sex (two categories). Finally, this study was not designed to ascertain whether there is a causal relationship between the observed trends and the COVID-19 pandemic, therefore longitudinal studies with larger samples would have been more adequate to establish causal relationships.

## Conclusions

Based on our study, it appears that suicide attempts among girls decreased during periods of strict confinement and school holidays, potentially indicating that reduced social and academic stressors initially benefited adolescent mental health. However, the subsequent increase in attempts, often associated with depression and social triggers, suggests that isolation measures adopted during the pandemic may have a more detrimental impact on the mental health of adolescents in the medium and long term, particularly affecting girls. The social and academic restriction measures have functioned akin to an anxiety avoidance mechanism, offering immediate relief by temporarily reducing stress. However, this approach hinders the development of adaptive and healthy coping skills, potentially leading to an increased sense of vulnerability. For adolescents, this heightened vulnerability could translate into a greater risk of suicide when confronted again with stress-inducing situations like returning to school and social interactions. Therefore, it is vital to explore new approaches that promote protective factors for adolescent mental health during future pandemics. This includes minimizing forced isolation while concurrently emphasizing training in coping strategies and social skills with peers. By taking these actions, we may be able to prevent mental disorders in this age group and reduce the risk of suicidal behavior.

## Data Availability

The data that support the findings of this study are available from corresponding authors upon reasonable request.
